# Progress in Cancer

**Published:** 1902-07-12

**Authors:** 


### Progress in Cancer.

(*Continued from page 243.*)

Three of Stanley Boyd's[5] list of cases of oophorectomy already published have remained free from recurrence for over 16 months. One case is well, 5 years 1 month; another (Dr. Herman's), 4 years 4 months, up to July 1901; no further note; the third case (Waterhouse) was well 2 years 4 months after operation. In other cases of the list growth was still left, and some had died; but these statistics compared favourably with Coley's mixed toxin treatment for sarcoma. The limitations of the operation would seem to be that it should be only used in cancer of the breast; the disease must be local, the extent being immaterial. Those cancers of slow growth give the best results. The prognosis is bad if there are obvious lesions in the viscera and bones. The menopause, though completely established, is not a contra-indication.

Simmons of Bangor[6] reports a case of a nullipara, aged about 40–45, in whose breast an ulcer presented of three years' malignant history. It seemed quite inoperable, and 19 months after this oöphorectomy was performed, and the ulcer of the breast, measuring $4\frac{1}{2}$ by $3\frac{1}{2}$ inches, with two surrounding nodules, but no obvious glandular infection, was completely healed over in eleven weeks from the date of operation by granulation. The nodules were softening at the time of report.

Paton,[7] on the other hand, records two cases of inoperable mammary scirrhus in patients with coincident pregnancy or lactation who received no benefit from oöphorectomy. While admitting the rise in the mortality of cancer, Geirsvold[8] points out that the disease has not yet been observed among Greenlanders, and is very rare in very cold climates, though not unknown in those parts of Norway within the Arctic circle. Cancer is more prevalent in towns, and more so in some than in others. He thinks there may be some connection between the hardness of the water and the incidence of the disease, the lime or magnesium salts having some irritating effect upon the mucus membrane of the stomach or intestines. For it is these organs and their accessory glands that are most affected. He finds that some localities are more affected than others, and that the higher classes show most cases, and the middle classes least.

Mitchell Banks[9] thinks that cancer is attacking people at a younger age than it used to do. He thinks one cause is the increased consumption of animal food. But, on the other hand, Pearce Gould[10] points out how even advanced cases of malignant disease disappear spontaneously. He looks for the future treatment along the lines of promoting the physiological processes which are occasionally noticed to be working spontaneously at present. He gives three cases of spontaneous cure.

Dr. Herbert Snow[11] quotes a case of recurrent inoperable carcinoma mammæ, with hundreds of secondary nodules on the skin from the scalp to the thighs. Morphia was administered and she improved, the nodules softened, and she lived for more than six years. Laurie Watson[12] records a case of a sarcoma of the back, which was removed but recurred immediately afterwards, ulcerated and became inoperable. Spontaneous improvement occurred, and 12 months after its first appearance it was very much smaller and absolutely painless.

The Röntgen rays have been applied with success by Peters[13] to a case of inoperable recurrent scirrhus. The patient was 93 years old, and 13 years previously Lord Lister had removed the left breast. Five years ago recurrence occurred in the scar and a nodule projected, adherent to the rib and $1\frac{1}{2}$ in. in diameter. It sloughed and progressed slowly, bleeding and pain being checked by suprarenal extract. Röntgen rays were then applied, the growth measuring $3 \times 2\frac{3}{4}$ in., with well-raised margin and irregular ulcerous centre. After ten applications at a distance of 2–4 in., for 10–20 minutes at a time, the growth has narrowed down to $1 \times \frac{3}{4}$ in., the margin being only slightly raised, and the central ulcer very small. Discharge and pain had decreased to a minimum. Unfortunately the patient died of intercurrent pneumonia, thus preventing the completion of what seemed to promise a cure.

According to Kakels[14] there are only three recorded cases of sarcoma of the pancreas, besides the one reported by himself, which was of the mixed celled variety. Malcolm[15] has removed, though unsuccessfully, a tumour of fibro-sarcomatous structure from the tail of the pancreas in a child aged four years eight months. Speaking of the use of arsenic and quinine in cancer, Benoit[16] holds that the former has an action unfavourable to the growth of cancer "germs." He urges the more frequent study of the drug, whether used hypodermically or internally. Quinine is reported to have caused great improvement when used hypodermically. He deprecates the use of Coley's fluid, of serums obtained from animals inoculated with cancer juice, or of nectrianine. He recommends the early free use of the knife, followed by the use of arsenic and quinine.

Coley[17] reports another case of good following the injection of his mixed toxins. The case was that of a man, 40 years of age, with a spindle cell sarcoma of the right superior maxilla. It could not be all removed, and he did badly after the operation. The liver also enlarged, and he became jaundiced. Injections of the toxins were given, mostly into the abdominal wall, and the patient began to put on weight, and recovered to resume his occupation, that of a veterinary surgeon.

In addition to a series of cases reported in the *Berliner klinische Wochenshrift*, No. 23, for 1901, Professor Albert Adamkiewicz[18] reports two cases successfully treated by means of his "cancroin." In the first of these cases, in June, 1900, there were cancerous tumours filling the entire true pelvis. In May, 1901, the patient was freed from the disease by means of the injections, and remained so until October, 1901, and in excellent health. The author lays stress upon the necessity that those conditions laid down by him must be combined in order to make the treatment a success. The second case was that of a married woman, aged 36, who was regarded as a hopeless case of carcinoma uteri by Chrobak in February, 1901. She soon became severely distressed by pain, dysuria, and pain and difficulty of defæcation, while intense vomiting made it difficult for her to retain any food. The cervix was widely ulcerated, with the growth involving the anterior wall of the rectum. "Cancroin" was injected, and hæmorrhage and abdominal pain disappeared promptly. She could eat with comfort, the discharges were passed without pain. The tumour on the anterior wall of the rectum disappeared, and in four weeks the patient walked freely about, "full of happiness and the enjoyment of life." Later, however, the injections were discontinued against advice, and intense pain, tenesmus and dysuria returned. On resuming the treatment, in four weeks she was in comfort again. Though a few painful contractions of the uterus, with dysuria and tenesmus, are still felt at times, yet the intervals are becoming longer as the treatment is continued, and she is in good health otherwise.

[5] Clin. Jour., March 19. [6] Med. Rec., March 15. [7] Brit. Med. Jour., March 1. [8] Treatment, Jan. [9] Lancet, March 22. [10] Lancet, March 22. [11] Clin. Jour., March 19. [12] Lancet, Feb. 1. [13] Brit. Med. Jour., March 1. [14] Amer. Jour. Med. Sci., March. [15] Lancet, March 1. [16] Treatment, Jan. [17] Med. Rec., Feb. 8. [18] Lancet, Feb. 1.

